# Effect of bisphosphonates in preventing femoral periprosthetic bone resorption after primary cementless total hip arthroplasty: a meta-analysis

**DOI:** 10.1186/s13018-015-0206-8

**Published:** 2015-05-13

**Authors:** Xinyu Zhao, Dongcai Hu, Jun Qin, Rahul Mohanan, Liaobin Chen

**Affiliations:** Department of Orthopaedics, Zhongnan Hospital of Wuhan University, 169 Donghu Road, Wuhan, Hubei Province 430071 China

**Keywords:** Total hip arthroplasty, Bisphosphonate, Bone mineral density, Meta-analysis

## Abstract

**Background:**

Bone loss leading to aseptic loosening of the prosthesis and periprosthetic fracture is a mode of failure in cementless total hip arthroplasty (THA). The aim of this meta-analysis was to evaluate the effect of bisphosphonates in preventing femoral periprosthetic bone resorption following primary cementless THA zone by zone.

**Method:**

Clinical randomized controlled trials concerning bisphosphonates application after primary cementless THA published up to October 2014 were retrieved from PubMed, Cochrane library, and Embase databases. The methodological quality of the included studies was assessed by the Physiotherapy Evidence Database (PEDro) scale. Data analysis was performed using StataSE12.0.

**Results:**

Ten randomized controlled trials involving a total of 502 patients were assessed; the bisphosphonates group included 256 patients and the control group included 246 patients. The meta-analysis showed that the bone mineral density (BMD) of most femoral periprosthetic zones in bisphosphonates group was significantly higher than that in the control group at 3 months postoperatively except zone 5 with no significant difference. At 6 and 12 months, the BMD of bisphosphonates group was much higher than that in control group except zone 5, which showed no statistical difference. The BMD of bisphosphonates group was persistently higher than control group in zone 6 and 7 at 5 years postoperatively, while the other zones had no significant difference. Both serum bone alkaline phosphatase and urinary type I collagen N-telopeptide were significantly suppressed by bisphosphonates at 3, 6, and 12 months.

**Conclusion:**

Bisphosphonates seem to decrease early femoral periprosthetic bone resorption after primary cementless THA. Drug efficacy was found to be long-standing in the main load-bearing zones.

## Background

Total hip arthroplasty (THA) is an effective treatment for end-stage avascular necrosis of femoral head, osteoarthritis, and rheumatoid arthritis of the hip [1]. By 2030, the demand for primary THA is estimated to reach 572,000, and the demand for hip revision is estimated to double in the USA [2]. Some studies indicated that more than 75 % of the revision arthroplasties were performed for aseptic loosening of the prosthesis and periprosthetic fracture, which were all found to be the sequel to severe periprosthetic bone loss [3].

Bisphosphonates are effective antiresorptive agents which have been used successfully to treat diseases characterized by osteoclast-mediated bone resorption, such as osteoporosis, Paget disease, and metastatic bone diseases [4]. Recently, several randomized controlled trials (RCTs) were performed to investigate the effect of bisphosphonates on femoral periprosthetic bone resorption following primary THA, most of which confirmed its efficacy, while some doubted it [5-7]. Two systematic reviews [8, 9] suggested that bisphosphonates have a beneficial effect on preserving periprosthetic bone in a short term after joint arthroplasty, while they had some limitations: (1) They ignored the fundamental difference between cement and cementless arthroplasty and did not describe it separately; The systematic reviews also included patients who underwent hemiarthroplasty and total knee arthroplasty and (2) They did not describe the periprosthetic bone loss zone by zone as they overlooked the uneven effect of bisphosphonates induced on different load-bearing areas of periprosthetic bone.

**Fig. 1:**
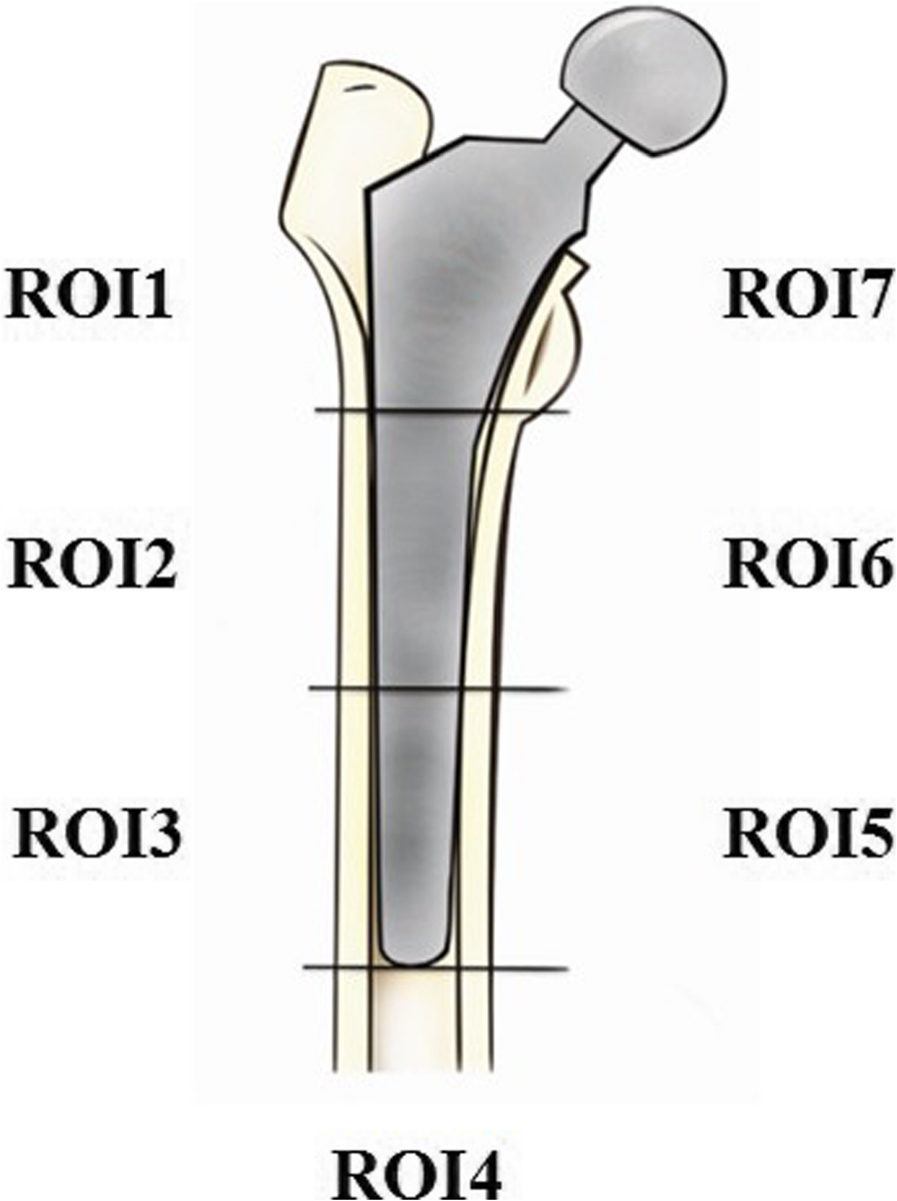
The seven regions of interest based on Gruen zones [10]

Therefore, we divided the femoral periprosthetic bone stock into seven regions of interest (ROI) as described by Gruen [10] (Fig. 1). We extracted the data of bone mineral density (BMD) from each included RCT, studying the effects of bisphosphonates in preventing femoral periprosthetic bone resorption following primary cementless THA. Serum bone alkaline phosphatase (BAP) and urinary type I collagen N-telopeptide breakdown products (NTX) were added as indices for the resorption. We performed the meta-analysis to clarify the effect of bisphosphonates in the treatment of periprosthetic bone resorption after cementless THA.

## Methods

### Search strategy

The PubMed, Embase, and Cochrane Central Register of Controlled Trials were searched from their earliest entries through October 2014. The search strategy was ((((random*[Title/Abstract]) OR “Randomized Controlled Trial” [Publication Type])) AND (((((hip arthroplasty [Title/Abstract]) OR hip replacement[Title/Abstract]) OR hip prosthesis[Title/Abstract])) OR “Arthroplasty, Replacement, Hip”[Mesh])) AND ((((((((risedronate[Title/Abstract]) OR tiludronate[Title/Abstract]) OR alendronate[Title/Abstract]) OR pamidronate[Title/Abstract]) OR etidronate[Title/Abstract]) OR zoledronate[Title/Abstract]) OR clodronate[Title/Abstract]) OR Bisphosphonate[Title/Abstract]). The reference list of the relevant literatures was also reviewed manually for any further relevant studies. Languages were not restricted in this search.

### Inclusion criteria and exclusion criteria

Inclusion criteria were as follows: (1) the target population consisted of patients undergoing primary cementless THA; (2) in the interventional group, the administration of the bisphosphonate group was oral, intramuscular, or intravenous, while the control group had been treated with calcium, alfacalcidol, or no medication; (3) the outcomes were analyzed with respect to BMD, serum BAP, and urinary NTX; and (4) the methodological criterion was prospective RCT.

Exclusion criteria were (1) cemented THA or other arthroplasties and (2) animal studies.

### Data extraction and assessment of methodological quality

After the consecutive procedures of screening of titles and abstracts, obtaining the full text of each article, and reviewing them, articles that met the eligibility criteria and did not meet the exclusion criteria were selected to be included. Data were extracted and collated independently by two authors (XYZ and DCH), including author, published year, sample size, patient age, sex, follow-up time, intervention protocol, BMD of each ROI in femoral periprosthetic bone, serum BAP, and urinary NTX. The data of a published updated study involving the same cohort of patients was extracted synthetically. The original investigators were contacted when requisite data were lacking in the publications. The methodological quality of each included RCT was assessed by two observers independently by the Physiotherapy Evidence Database (PEDro) scale [11], and trials with a score of 6 or more were considered high quality. Disagreements were resolved by means of discussion with the corresponding author (LBC).

### Statistical methods

The meta-analysis was conducted with StataSE12.0 software. The weighted mean difference (WMD) and 95 % confidence interval (CI) were calculated for continuous data, and the relative risk (RR) and 95 % CI were calculated for dichotomous data. The statistical heterogeneity was tested with the chi-square test and *I*^2^. If heterogeneity was low (*P* > 0.1, *I*^2^ < 50 %), a fixed-effect model was used. If heterogeneity was significant (*P* < 0.1 ,*I*^2^ > 50 %), sensitivity analysis, subgroup analyses, and meta-regression were conducted to find the source of the heterogeneity. If the heterogeneity could not be eliminated, a random-effect model would be used when the result of meta-analysis had clinical homogeneity, or descriptive analysis would be used.

## Results

### Study characteristics

**Fig. 2:**
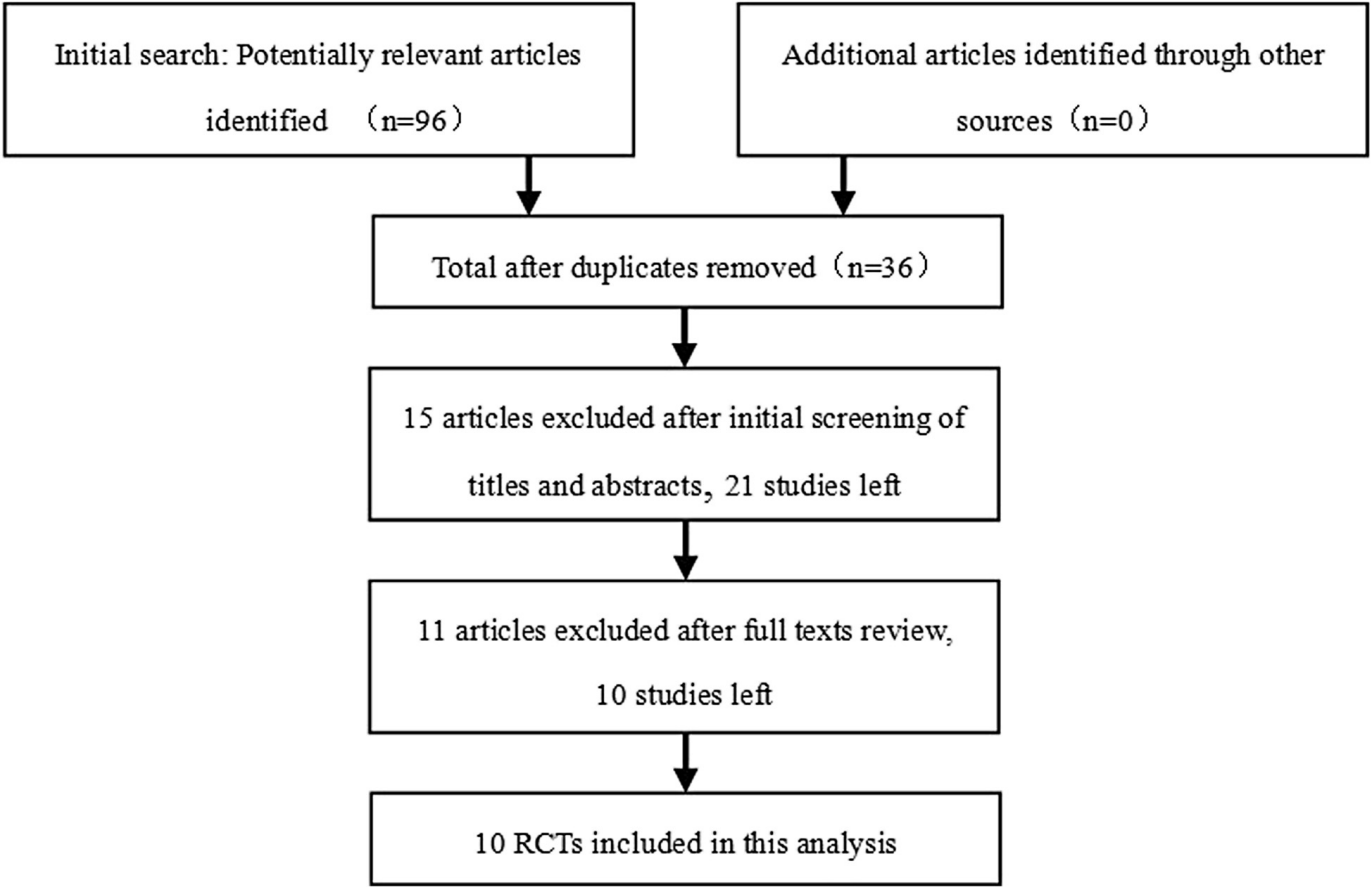
Flow chart summarizing the selection process of randomized control trials

A total of 96 potential articles were identified and screened for the meta-analysis. After screening of titles and abstracts, obtaining the full text of each article, and reviewing them, ten RCTs were selected for this meta-analysis [7, 12-20] (Fig. 2). The cumulative sample size of 502 primary cementless THA comprised 256 with bisphosphonates and 246 without bisphosphonates. The main characteristics of the included studies were summarized in Table 1 and the literature-exclusion procedure was depicted in Fig. 2. The methodological quality of the included RCTs was assessed with the PEDro scale (Table 2), the results showed that all RCTs were of high quality.

**Table 1:** Details on the included studies in the meta-analysis

Author	Year of publication	Follow-up time (months)	Sample size	Patient mean age (years)	Sex	Bisphosphonates/control group	Intervention protocol
Hennigs T	2002	12	66	51.5	29/27	42/24	Subgroup 1: oral alendronate 10 mg/day for 10 weeks
							Subgroup 2: oral alendronate 20 mg/day for 5 weeks
Arabmotlagh M	2006	72	51	62.5	26/25	27/24	Subgroup 1: oral alendronate 20 mg/day for 2 months, thereafter 20 mg/day for 4 months
							Subgroup 2: oral alendronate 20 mg/day for 2 months, thereafter 20 mg/day for 6 months
Arabmotlagh M	2009	72	49	62.5	25/24	29/20	Subgroup 1: oral alendronate 10 mg/day for 10 weeks
							Subgroup 2: oral alendronate 20 mg/day for 5 weeks
Iwamoto N	2011	12	60	65	14/46	20/40	Oral alendronate 5 mg/day for 48 weeks
Skoldenberg OG	2011	24	73	60	30/43	36/37	Oral risedronate 35 mg/week for 6 months
Tapaninen TS	2010	60	16	61.4	7/9	7/9	Oral alendronate 10 mg/day for 6 months
Trevisan C	2010	12	91	64.7	53/58	42/49	Oral clodronate 100 mg/day for 10 days, thereafter 100 mg/week for 50 weeks
Venesmaa PK	2001	6	13	62.62	6/7	8/5	Oral alendronate 10 mg/day for 6 months
Yamaguchi K	2005	12	43	68.5	0/44	26/17	Subgroup 1: oral etidronate 200 mg/day for 2 weeks, followed by 12 weeks of calcium lactate of 500 mg/day, the cycle was repeated four times
							Subgroup 2: oral etidronate 400 mg/day for 2 weeks, followed by 12 weeks of calcium lactate of 500 mg/day, the cycle was repeated four times
Yamasaki S	2007	6	40	66.7	4/36	19/21	Oral risedronate 2.5 mg/week for 6 months

**Table 2:** PEDro critical appraisal scores

Author	PEDro critical appraisal score											Total
	1	2	3	4	5	6	7	8	9	10	11	
Hennigs T 2002	Y	Y	Y	Y	Y	Y	N	Y	Y	Y	Y	9
Arabmotlagh M 2006	Y	Y	Y	Y	Y	Y	N	Y	Y	Y	Y	9
Arabmotlagh M 2009	Y	Y	Y	Y	Y	Y	N	Y	Y	Y	Y	9
Iwamoto N 2011	Y	Y	Y	Y	Y	N	N	Y	Y	Y	Y	8
Skoldenberg OG 2011	Y	Y	Y	Y	Y	Y	N	Y	Y	Y	Y	9
Tapaninen TS 2010	Y	Y	N	Y	N	N	N	Y	Y	Y	Y	6
Trevisan C 2010	Y	Y	N	Y	N	N	N	Y	Y	Y	Y	6
Venesmaa PK 2001	Y	Y	Y	Y	N	N	N	Y	Y	Y	Y	7
Yamaguchi K 2005	Y	Y	N	Y	N	N	N	Y	Y	Y	Y	6
Yamasaki S 2007	Y	Y	N	Y	Y	N	N	Y	Y	Y	Y	7

PEDro criteria: (1) eligibility criteria, (2) random allocation, (3) concealed allocation, (4) baseline comparability, (5) participant blinding, (6) therapist blinding, (7) assessor blinding, (8) >85 % follow-up, (9) intention-to-treat analysis, (10) between-groups statistical comparison for at least one key outcome, and (11) point estimates and variability measures for at least one key outcome. A trial with a score of 6 or more was considered high quality.

### Mean percentage changes of BMD in femoral periprosthetic ROI

The BMD was assessed by the method of dual-energy X-ray absorptiometry in all the included RCTs. The changes of BMD in femoral periprosthetic ROI at 3 months after surgery were reported in four [12, 13, 16, 17] of the ten studies. The results of meta-analysis of some ROI appeared heterogeneous, and sensitivity analysis indicated that the heterogeneity came from the studies of Skoldenberg et al. [13] and Trevisan et al. [17]. Subgroup analyses failed to eliminate the heterogeneity, then we found that, regardless of the exclusion or inclusion of these two studies, the results of meta-analysis were all the same and had clinical agreement, so we included these two studies and conducted the meta-analysis by random-effect model for the reason that these two studies were of high quality. Meta-analysis indicated that BMD ratios in the bisphosphonates group were significantly higher than those in the control group mainly in zones 1, 2, 3, 4, 6, and 7 (*P* < 0.05), while lower in zone 5 (WMD = −0.619, 95 % CI: −1.120 ~ −0.119, *P* < 0.015, Table 3).

**Table 3:** Comparison of postoperative BMD ratios at 3 months between each group

ROI	BMD ratios in the bisphosphonates group	BMD ratios in the control group	WMD	[95 % CI]		*P* of chi-square	*I* ^2^	Selected model	*P* for overall effect
1	97.556	90.840	6.364	2.122	10.606	0	92 %	Random-effect model	0.003
2	96.275	93.488	2.479	1.886	3.072	0.564	0 %	Fixed-effect model	<0.001
3	97.041	95.413	1.309	0.824	1.794	0.673	0 %	Fixed-effect model	<0.001
4	100.207	98.036	2.349	0.505	4.193	0.003	78 %	Random-effect model	0.013
5	97.528	97.172	−0.619	−1.120	−0.119	0.138	46 %	Fixed-effect model	0.015
6	95.641	92.724	2.177	1.598	2.756	0.552	0 %	Fixed-effect model	<0.001
7	91.099	84.457	6.634	0.655	12.612	0.001	95 %	Random-effect model	0.030

The changes of BMD in femoral periprosthetic ROI at 6 months after surgery were reported in eight [7, 12-17, 20] of the ten studies. Heterogeneity was analyzed through the same method, and random-effect model would be used if necessary. The pooling result showed that the BMD of the bisphosphonates group in most zones were significantly higher than that of the control group (*P* < 0.05, Table 4) except no statistical difference of BMD in zone 5 (WMD = 0.564, 95 % CI: −0.855 ~ −1.983, *P* = 0.436).

**Table 4:** Comparison of postoperative BMD ratios at 6 months between each group

ROI	BMD ratios in the bisphosphonates group	BMD ratios in the control group	WMD	[95 % CI]		*P* of chi-square	*I* ^2^	Selected model	*P* for overall effect
1	98.035	89.227	8.422	4.313	12.531	0.001	88 %	Random-effect model	<0.001
2	97.881	93.224	4.142	0.611	7.672	0.001	85 %	Random-effect model	0.021
3	97.974	95.954	1.930	0.668	3.193	0.041	52 %	Random-effect model	0.003
4	99.810	98.305	1.357	0.188	2.525	0.039	53 %	Random-effect model	0.023
5	99.638	98.188	0.564	−0.855	1.983	0.015	59 %	Random-effect model	0.436
6	96.283	92.148	3.863	1.291	6.435	0.001	80 %	Random-effect model	0.003
7	87.788	79.417	8.371	4.239	12.503	0.001	86 %	Random-effect model	<0.001

Six papers [12, 13, 15, 17, 19, 20] described the postoperative BMD ratios at 12 months, and the same procedure of analysis was performed. The Forest plots also indicated that the BMD of the bisphosphonates group were significantly higher (*P* < 0.05, Table 5) than that of the control group except zone 5 with no statistical difference (*P* = 0.696, Table 5).

**Table 5:** Comparison of postoperative BMD ratios at 12 months between each group

ROI	BMD ratios in bisphosphonates group	BMD ratios in control group	WMD	[95 % CI]		*P* of chi-square	*I* ^2^	Selected model	*P* for overall effect
1	96.005	87.601	6.819	3.816	9.821	0.009	67 %	Random-effect model	<0.001
2	99.032	92.431	6.250	5.314	7.186	0.386	8 %	Fixed-effect model	<0.001
3	99.786	97.172	2.822	0.615	5.029	0.001	86 %	Random-effect model	0.012
4	101.002	98.085	2.540	0.948	4.132	0.002	73 %	Random-effect model	0.002
5	101.189	100.601	−0.303	−1.823	1.218	0.074	50 %	Fixed-effect model	0.696
6	97.458	91.817	5.220	3.262	7.178	0.018	63 %	Random-effect model	<0.001
7	85.797	77.128	8.153	5.130	11.175	0.002	73 %	Random-effect model	<0.001

The postoperative BMD ratios at 5 years after surgery were calculated in two studies [7, 19], and fixed-effect model was used in all zones as no heterogeneity was detected. The results of meta-analysis showed that BMD of the bisphosphonates group in zones 6 and 7 were significantly higher (*P* < 0.05) than that of the control group, while no statistical difference were found in the rest of the zones (*P* > 0.05, Table 6).

**Table 6:** Comparison of postoperative BMD ratios at 5 years between each group

ROI	BMD ratios in bisphosphonates group	BMD ratios in control group	WMD	[95 % CI]		*P* of chi-square	*I* ^2^	Selected model	*P* for overall effect
1	98.301	93.225	3.113	−7.234	13.461	0.364	0 %	Fixed-effect model	0.555
2	95.222	93.382	−0.391	−6.359	5.578	0.387	0 %	Fixed-effect model	0.898
3	95.092	96.161	−2.885	−6.893	1.123	0.508	0 %	Fixed-effect model	0.158
4	95.685	96.943	−2.756	−6.559	1.047	0.640	0 %	Fixed-effect model	0.155
5	95.570	96.829	−1.618	−5.633	2.398	0.750	0 %	Fixed-effect model	0.430
6	98.976	92.007	7.002	0.004	14.001	0.925	0 %	Fixed-effect model	0.050
7	79.444	69.561	9.664	1.754	17.575	0.852	0 %	Fixed-effect model	0.017

### Serum bone alkaline phosphates

**Fig. 3:**
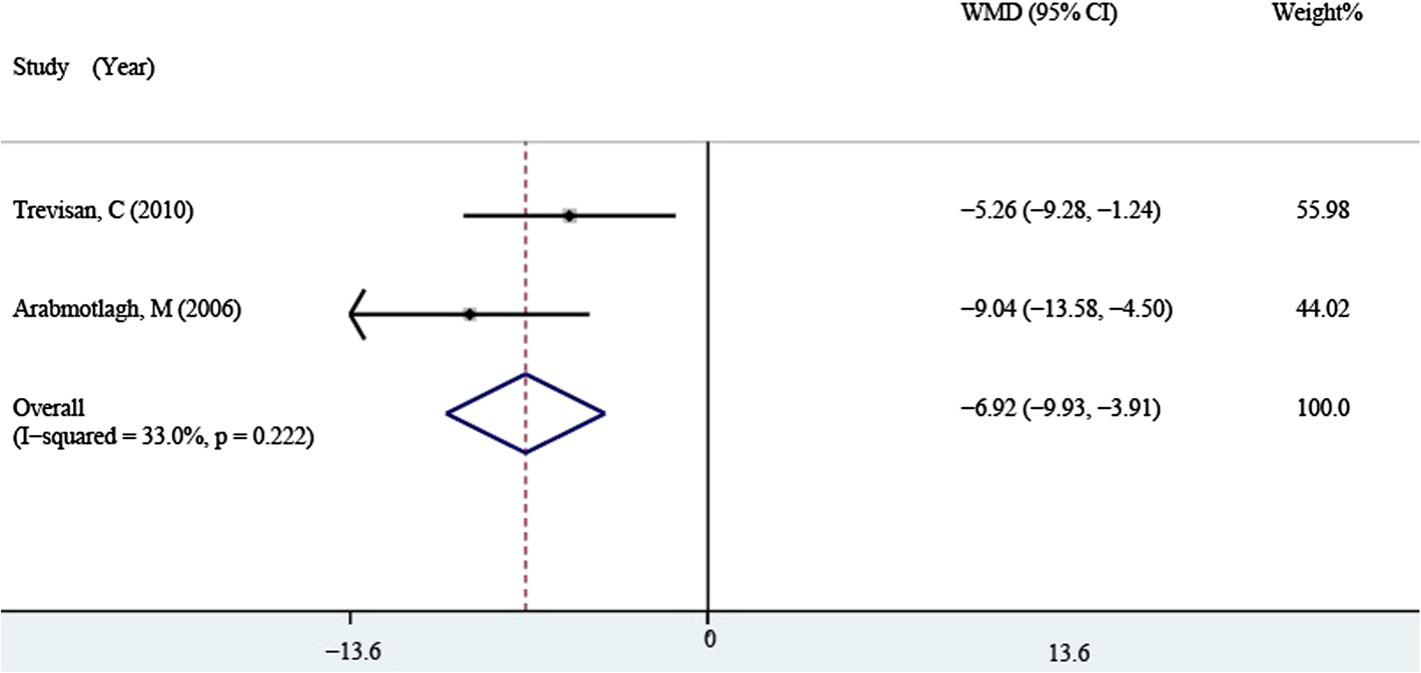
Comparison of serum BAP between the bisphosphonate and control group at 3 months

Two papers [17, 18] including 71 patients in the bisphosphonates group and 69 patients in the control group described the postoperative serum BAP at 3 months, fixed-effect model was used as heterogeneity was not detected (*P* = 0.222, *I*^2^ = 33). The pooling result showed the serum BAP in the bisphosphonates group was significantly lower than that of the control group (WMD = −6.924, 95 % CI: −9.934 ~ −3.913, *P* = 0.001. Fig. 3).

**Fig. 4:**
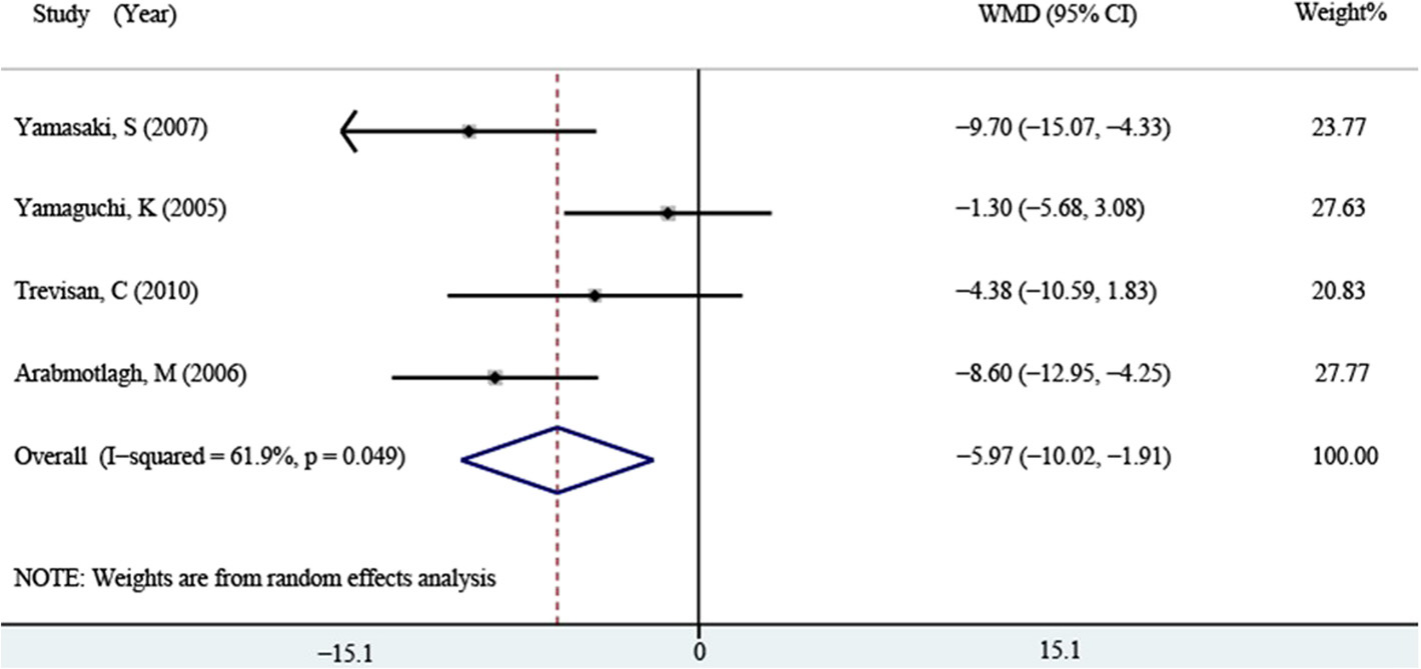
Comparison of serum BAP between the bisphosphonate and control group at 6 months

The serum BAP at 6 months after surgery was reported in four [14, 15, 17, 18] of the ten studies, including 116 patients in the bisphosphonates group and 107 patients in the control group, and the same procedure of analysis was performed and random-effect model was used (*P* = 0.049, *I*^2^ = 61.9 %). The results of meta-analysis indicated that serum BAP of the bisphosphonates group was significantly lower than that of the control group (WMD = −5.874, 95 % CI: −8.332 ~ −3.416, *P* = 0.001, Fig. 4).

**Fig. 5:**
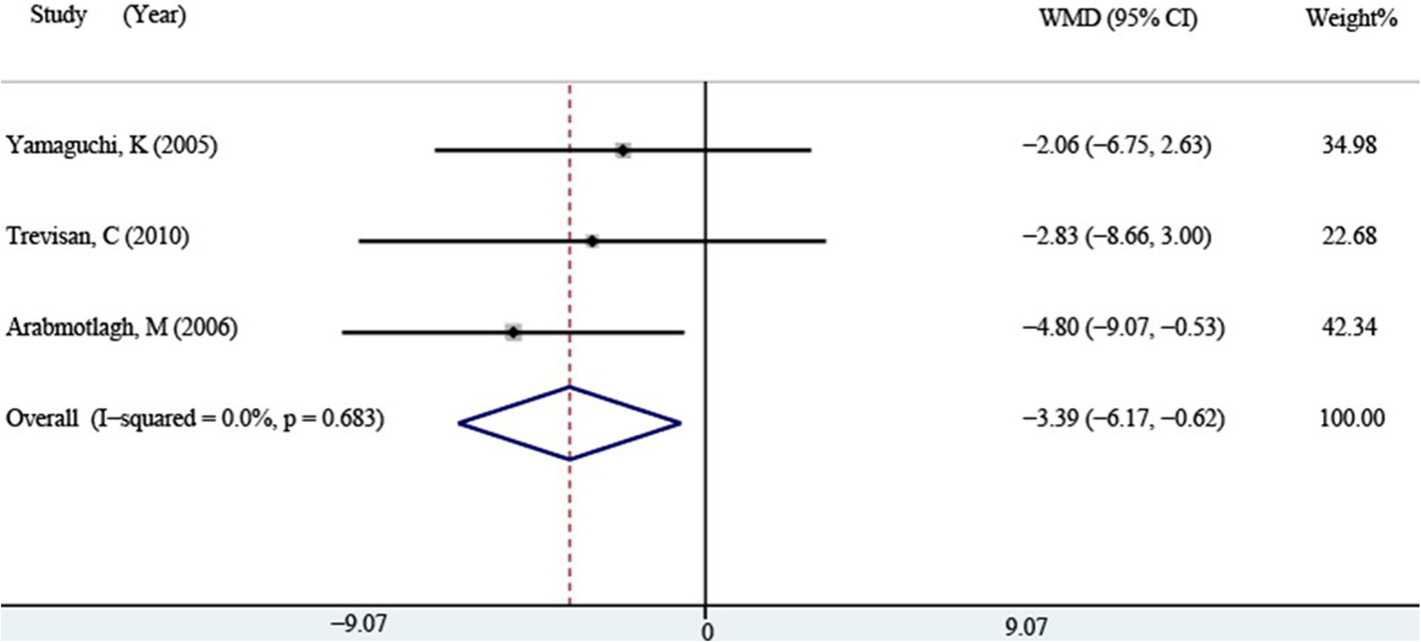
Comparison of serum BAP between the bisphosphonate and control group at 12 months

Three studies [15, 17, 18] including 95 patients in the bisphosphonates group and 86 patients in the control group described the postoperative serum BAP at 12 months. The Forest plots of fixed-effect model (*P* = 0.683, *I*^2^ = 0 %) also indicated that serum BAP of the bisphosphonates group was significantly lower than that of the control group (WMD = −3.395, 95 % CI: −6.171 ~ −0.619, *P* = 0.017, Fig. 5).

### Urinary type I collagen N-telopeptide

**Fig. 6:**
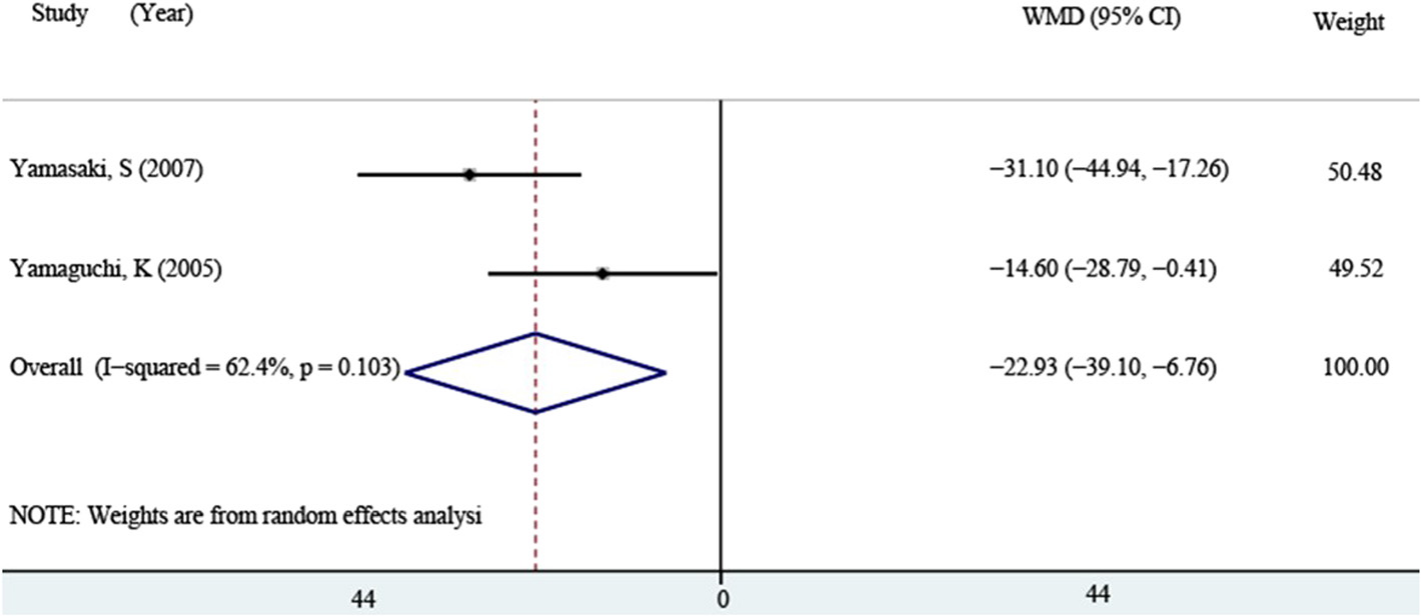
Comparison of urinary NTX between the bisphosphonate and control group at 6 months

The urinary NTX at 6 months after surgery was reported in two [14, 15] of the ten studies, including 45 patients in the bisphosphonates group and 38 patients in the control group, and random-effect model was used (*P* = 0.049, *I*^2^ = 61.9 %). The pooling result indicated that urinary NTX of the bisphosphonates group was significantly lower than that of the control group (WMD = −22.929, 95 % CI: −39.098 ~ −6.760, *P* = 0.005, Fig. 6).

Yamasaki et al. [14] reported that the urinary NTX in the bisphosphonates group at 12 months after surgery was lower than that of the control group.

## Discussion

THA is an effective treatment for end-stage hip disease, but the aseptic loosening of implants and the periprosthetic fracture secondary to periprosthetic bone loss remain an unresolved problem. Three mechanisms are thought to contribute to femoral periprosthetic bone resorption [21]. (1) The intraoperative mechanical, thermal, and chemical damage cause necrosis in bone stock of variable size. It might take approximately 3 months to heal; (2) Delayed bone resorption process called ‘stress shielding’ occurs in proximal regions of the femur, which is related to the biomechanical characteristics of the bone-implant structure and the difference in stiffness of the prosthesis compared to the surrounding bone. Stress shielding tends to stabilize by 1 year postoperatively. (3) The inflammatory response caused by the detritus produced by the wear and tear of prosthesis is another reason for osteolysis, which mainly happens 5 years after surgery. If an ideal drug suppressing the bone resorption after THA was found, the service life of prosthesis would be much prolonged by maintaining BMD around it [21]. The antiresorptive effect of bisphosphonate is cell mediated, mainly by direct inhibitory effect on osteoclastic recruitment [18, 21, 22]. For patients after THA, the administration of bisphosphonates may decrease the risk of future hip fractures, reduce the chances of subsidence of the stem, lower the risk of revision, and prolong the survival time of prosthesis; however, some studies still doubt its long-term efficacy [13, 23-25].

The results of our meta-analysis indicated that the periprosthetic BMD in the bisphosphonate group was higher than that of the control group in most areas at 3, 6, and 12 months postoperatively. This effect seemed to persist in the main load-bearing areas of zones 6 and 7 at 5 years postoperatively. These results suggest that bisphosphonates decrease early femoral periprosthetic bone resorption after primary cementless THA and their efficacy are long-standing in the main load-bearing zones. Then, we discussed the changes of BMD combined with the alterations of biomechanical characteristic in the proximal-medial area of the femur after THA. The normal mechanical loading in proximal femur starts in zone 7, then goes down to the cortical bone of zone 6. After turning to zone 3, the mechanical loading will continue downward. The cortical bone of zones 1 and 2 also shares part of the loading. After THA, the stress shielding will cause significant decrease in load bearing in zones 1, 2, 3, 6, and 7. However, the load bearing in zone 5 does not change apparently [26, 27]. In our study, the pooling results at 3 months after surgery indicated the BMD of the bisphosphonates group was all significantly higher than that of the control group except zone 5. The reason for this phenomenon might be that the bone resorption due to stress shielding was significantly suppressed by bisphosphonates in most zones; however, stress shielding is not apparent in zone 5, which limits the antiresorptive efficacy of bisphosphonates [26, 27]. As the accumulation of bone formation, no difference was detected in zone 5 at 6 and 12 month after surgery. After 3 months, the reconstruction of the intraoperative bone damage has finished and the bone resorption induced by stress shielding becomes the leading effect [12, 13, 19, 28], so the BMD in the bisphosphonates group were found to be higher than that of the control group except zone 5. At the fifth postoperative year, by pooling the results of the existing studies, we found that the BMD of the main load-bearing zones 6 and 7 in the bisphosphonates group was still higher than that of the control group, which suggests that the efficacy of limited course of bisphosphonates in the existing studies are long-standing in the main load-bearing zones.

The results of our meta-analysis showed that the serum BAP and urinary NTX were suppressed by bisphosphonates in postoperative 1 year, which also proves the antiresorptive efficacy of bisphosphonates from metabolic level.

Only one of our included studies had a course of bisphosphonates lasting for 1 year, the rest were all less than 6 months, and all follow-up were less than 6 years. Eberhardt et al. [29] reported that postoperative continuous and high-dose bisphosphonate treatment is potent in accelerating osseointegration of the prosthesis, which may prevent wear debris from migration by sealing the implant-bone interface. However, the efficacy of bisphosphonates on this phenomenon needs further research as the limited course of treatment and follow-up in our study. The current studies suggest that the timing of the administration of bisphosphonate may be related to its efficacy [6, 16, 18]; a long-term administration of bisphosphonate is well tolerated and can increase BMD remarkably [30]. Considering that bone loss around the prosthesis of THA is suspected to be progressive and faster than that due to normal aging, Skoldenberg et al. [13] suggested that the duration of bisphosphonate treatment should be lifelong to achieve a reduced risk of revision and an improved quality of life; however, this hypothesis needs further research.

The limitations of our study include the following. (1) The BMD results of meta-analysis of some ROI appeared heterogeneous, and sensitivity analysis and subgroup analyses failed to eliminate the heterogeneity. As those results of meta-analysis had clinical agreement, we included those studies and conducted the meta-analysis by random-effect model for the reason that those studies were of high quality, which may slightly influence the reliability of the meta-analysis. (2) The included studies did not have sufficient duration of bisphosphonate treatment and follow-up, and they also lacked evaluating indexes like functional scores and the rate of revision, so we could not evaluate the efficacy of postoperative bisphosphonate treatment comprehensively.

## Conclusion

Bisphosphonates seem to decrease early femoral periprosthetic bone resorption after primary cementless THA, their efficacy are long-standing in the main load-bearing zones. Considering the conclusions of other researches and the fact that most of our included studies just had a course of bisphosphonates treatment for less than 6 months, the long-term effects should be evaluated by new RCTs, which should be performed with a longer duration of bisphosphonates administration and follow-up to clarify the best dosage and duration of bisphosphonates treatment.

## Abbreviations

THA: Total hip arthroplasty
PEDro: Physiotherapy Evidence Database
BMD: Bone mineral density
RCTs: Randomized controlled trials
ROI: Regions of interest
BAP: Bone alkaline phosphatase
NTX: Urinary type I collagen N-telopeptide breakdown products
WMD: Weighted mean difference
CI: Confidence interval
